# Solanaceous exocyst subunits are involved in immunity to diverse plant pathogens

**DOI:** 10.1093/jxb/erx442

**Published:** 2018-01-10

**Authors:** Yu Du, Elysa J R Overdijk, Jeroen A Berg, Francine Govers, Klaas Bouwmeester

**Affiliations:** 1 College of Horticulture, Northwest A&F University, Yangling, Shaanxi, China; 2 Laboratory of Phytopathology, Wageningen University & Research, Wageningen, The Netherlands; 3 Laboratory of Cell Biology, Wageningen University & Research, Wageningen, The Netherlands

**Keywords:** Basal defence, exocyst complex, exocytosis, gene silencing, phylogenetic analysis, plant resistance, Solanaceous plants, vesicle trafficking

## Abstract

The exocyst, a multiprotein complex consisting of eight subunits, plays an essential role in many biological processes by mediating secretion of post-Golgi-derived vesicles towards the plasma membrane. In recent years, roles for plant exocyst subunits in pathogen defence have been uncovered, largely based on studies in the model plant Arabidopsis. Only a few studies have been undertaken to assign the role of exocyst subunits in plant defence in other plants species, including crops. In this study, predicted protein sequences from exocyst subunits were retrieved by mining databases from the Solanaceous plants *Nicotiana benthamiana*, tomato, and potato. Subsequently, their evolutionary relationship with Arabidopsis exocyst subunits was analysed. Gene silencing in *N. benthamiana* showed that several exocyst subunits are required for proper plant defence against the (hemi-)biotrophic plant pathogens *Phytophthora infestans* and *Pseudomonas syringae*. In contrast, some exocyst subunits seem to act as susceptibility factors for the necrotrophic pathogen *Botrytis cinerea.* Furthermore, the majority of the exocyst subunits were found to be involved in callose deposition, suggesting that they play a role in basal plant defence. This study provides insight into the evolution of exocyst subunits in Solanaceous plants and is the first to show their role in immunity against multiple unrelated pathogens.

## Teaser

This study provides insight into the evolution of the exocyst subunits of Solanaceous plants and is the first to show their role in immunity against multiple unrelated pathogens.

## Introduction

The exocyst, an evolutionary conserved multiprotein complex that consists of eight subunits, is involved in directing post-Golgi-derived vesicles towards the plasma membrane (Munson and Novick, 2006). This process works one step before docking and fusion of vesicles at the membrane mediated by the soluble SNARE complex (Duman and Forte, 2003). The exocyst complex was first discovered in yeast and shown to be required for secretion and polarized growth during budding (Terbush and Novick, 1995). Later on, homologues of the eight exocyst subunits, namely Sec3, Sec5, Sec6, Sec8, Sec10, Sec15, Exo70, and Exo84, were identified in higher organisms (Ting *et al.*, 1995; Hsu *et al.*, 1996; Kee *et al.*, 1997; Brymora *et al.*, 2001; Matern *et al.*, 2001). In yeast and mammals, interaction between the individual exocyst subunits was studied by different methods, and this revealed that each exocyst subunit can associate with multiple other subunits in the complex (Munson and Novick, 2006). Studies using chromatographic fractionation and yeast two-hybrid assays showed that also in plants the exocyst is a true protein complex and that the pairwise interactions between the exocyst subunits are conserved, resembling those in yeast and animals (Hála *et al.*, 2008; Zhang *et al.*, 2010; Žárský *et al.*, 2013; Zhang *et al.*, 2013). Unlike yeast and animals, which have single-copy genes for each exocyst subunit, plants often have two or more genes encoding the same subunit, for example Arabidopsis has three Exo84 and 23 Exo70 paralogues (Synek *et al.*, 2006; Chong *et al.*, 2010; Cvrčková *et al.*, 2012).

So far, several studies have shown that exocyst subunits have a role in plant growth and development. For example, multiple exocyst subunits were reported to be required for proper cytokinesis (Fendrych *et al.*, 2010; Rybak *et al.*, 2014). It was also suggested that the exocyst complex is crucial for polar exocytosis during growth of pollen and roots (Cole *et al.*, 2005, 2014; Synek *et al.*, 2006; Hála *et al.*, 2008; Bloch *et al.*, 2016; Tan *et al.*, 2016). Furthermore, studies showed that the exocyst complex is required for a broad variety of developmental processes, including pollen acceptance (Samuel *et al.*, 2009; Safavian *et al.*, 2015), secondary cell wall deposition (Li *et al.*, 2013; Kulich *et al.*, 2015; Oda *et al.*, 2015; Vukašinović *et al.*, 2017), PIN protein recycling (Drdová *et al.*, 2013; Tan *et al.*, 2016), stomatal opening (Hong *et al.*, 2016; Seo *et al.*, 2016), and autophagy (Kulich *et al.*, 2013).

In plants, vesicle trafficking is an integral component of the immune mechanism and plays an important role in defence against pathogens by accommodating secretion of antimicrobial compounds to infection sites to hamper pathogen colonization. For example, infection of barley with the powdery mildew fungus *Blumeria graminis* leads to a cell wall-associated defence that is accompanied by accumulation of multivesicular bodies that participate in cell wall apposition, and reactive oxygen species (ROS) accumulation at the cell periphery (An *et al.*, 2006). In addition, penetration resistance of Arabidopsis to *B. graminis* was shown to be mediated by the syntaxin SYP121, a component of the SNARE complex (Collins *et al.*, 2003). Moreover, induction of systemic acquired resistance was reported to be dependent on NPR1-regulated expression of several secretion-related genes (Wang *et al.*, 2005).

Emerging evidence shows that the exocyst complex is an additional key player in plant–pathogen interactions. This is mostly based on studies that use Exo70 mutant plants (Martin-Urdiroz *et al.*, 2016). Arabidopsis *Exo70B1* mutants show lesion-mimic cell death mediated by salicylic acid accumulation (Kulich *et al.*, 2013; Stegmann *et al.*, 2013). Arabidopsis *Exo70B1* is required for autophagy, a process involved in degradation and recycling of cellular components, and plays an important role in receptor signalling during plant defence (Kulich *et al.*, 2013). Zhao *et al.* (2015) showed that Exo70B1 interacts with TIR-NBS2, an atypical nucleotide-binding domain and leucine-rich repeat-containing (NLR) resistance protein that lacks the leucine-rich repeat domain, and that disruption of this interaction leads to constitutive activation of defence. Recently, Sabol *et al.* (2017) showed that Arabidopsis Exo70B1 also interacts with RIN4, a well-known regulator of plant defence that is cleaved by the bacterial protease AvrRpt2 of *Pseudomonas syringae* (Afzal *et al.*, 2011). Hence, it was suggested that cleavage of RIN4 releases Exo70B1 from the plasma membrane and thereby suppresses tethering of defence-related vesicles (Sabol *et al.*, 2017). Barley Exo70F-like, and Arabidopsis Exo70B2 and Exo70H1 were reported to be involved in basal resistance against *B. graminis* (Pečenková *et al.*, 2011; Ostertag *et al.*, 2013). Arabidopsis Exo70B2—the target of the ubiquitin ligase PUB22—was found to be required for full activation of immune signalling elicited by pathogen-associated molecular patterns (PAMPs) (Stegmann *et al.*, 2012). Pečenková *et al.* (2011) found that Arabidopsis *Exo70B2* mutants develop aberrant papillae with halos and show enhanced penetration by *B. graminis*. Besides Exo70B2, the SNARE syntaxin PENETRATION1 (PEN1) is also required for timely assembly of papillae and focal secretion, and thus for blocking penetration by *B. graminis* (Collins *et al.*, 2003). Penetration resistance is often conferred by cell wall thickening, of which callose is a major component (Collinge, 2009). In *Nicotiana benthamiana*, silencing of *Sec5* impairs callose deposition (Du *et al.*, 2015) and, in Arabidopsis, Exo70H4 was found to be important for callose deposition in trichomes (Kulich *et al.*, 2015). As such it can be hypothesized that the exocyst is mediating callose deposition at infection sites. Exocyst subunits were also reported to play an important role during arbuscular mycorrhizal symbiosis, suggested by their accumulation at sites of microbial contact and role in the formation of the perifungal membrane (Genre *et al.*, 2012; Zhang *et al.*, 2015).

The importance of vesicle trafficking and exocytosis in plant immunity was also shown by the fact that plant pathogens secrete effectors to target vesicle trafficking-related proteins. Examples are the bacterial pathogen *P. syringae* that uses its effector HopM1 to destabilize AtMIN7—a key regulator of vesicle formation—to facilitate infection of Arabidopsis (Nomura *et al.*, 2006, 2011), and the fungal plant pathogen *Alternaria carthami* that secretes the phytotoxin brefeldin A (BFA) to inhibit Golgi-derived vesicle formation (Driouich *et al.*, 1997). Furthermore, it was shown that the effector AVR-Pii from *Magnaporthe oryzae* targets rice Exo70F2 and -F3 (Fujisaki *et al.*, 2015), and that the oomycete *Phytophthora infestans* exploits the RXLR effector AVR1 for targeting the exocyst subunit Sec5 in potato to suppress host defence (Du *et al.*, 2015).

In general, the role of the exocyst complex in plant defence has not been studied in depth. The knowledge obtained so far is rather fragmented and covers only a few of the Exo70 paralogues and two other exocyst subunits, namely Sec5 and Exo84B. The aim of this study was to investigate the role of exocyst subunits in plant immunity of the Solanaceous model plant *N. benthamiana*. Genes encoding the exocyst subunits were identified in Solanaceous plants and phylogenetic analyses were performed to study their relationship with exocyst subunits of Arabidopsis. We then employed *Tobacco rattle virus* (TRV)-mediated virus-induced gene silencing (VIGS) in *N. benthamiana* and tested the effect of silenced exocyst subunit genes in disease assays. The results show that multiple Solanaceous exocyst subunits play a role in defence against plant pathogens with different lifestyles.

## Materials and methods

### Gene identification and phylogenetic analysis

Protein sequences of the *Arabidopsis thaliana* exocyst subunits were retrieved from the TAIR database using the gene inventory of Chong *et al.* (2010). BLAST analysis was subsequently performed against the genomes and predicted proteomes of *N. benthamiana*, *Solanum tuberosum* (potato), and *Solanum lycopersicum* (tomato) at the Sol Genomics Network (SGN) website (http://solgenomics.net). Protein sequences are listed in Supplementary Table S1 at *JXB* online.

Protein sequence alignments were constructed using ClustalW with default settings (protein weight matrix GONNET, gap opening of 10, gap extension of 0.2). Obtained sequence alignments were used as input to reconstruct phylogenetic trees using the Neighbor–Joining algorithm in MEGA5 with 5000 bootstrap replicates. Branches corresponding to partitions reproduced in <50% of bootstrap replicates were collapsed.

### Plasmid construction

Multisequence alignments by ClustalW or MultAlin were used to pinpoint gene segments containing stretches of >25 nucleotides with 100% identity to the target gene. The specificity of gene silencing was verified by BLAST analysis and the VIGS web-tool at the SGN website. Gene segments containing either *Eco*RI/*Sac*I or *Bam*HI/*Sac*I restriction sites were synthesized by Eurofins Genomics (see Supplementary Table S2), and cloned into the binary vector pTRV2. Binary plasmids were transformed to *Agrobacterium tumefaciens* strain AGL1 via electroporation. TRV constructs were generated to silence the *N. benthamiana Sec* and *Exo* genes in the various clades and subclades (Supplementary Table S2). Four of the silencing constructs contain multiple target sequences in order to silence all genes in the subclades Exo70C, Exo70D, Exo70G, and Exo70H, respectively.

### Plant material and pathogen growth

*Nicotiana benthamiana* was grown in potting soil under standardized greenhouse conditions. *Phytophthora infestans* isolate 14-3-GFP was grown in the dark on rye sucrose agar medium at 18 °C. *P. infestans* zoospores were isolated according to Champouret *et al.* (2009) and the concentration was adjusted to 1 × 10^5^ zoospores ml^–1^. *N. benthamiana* leaves were detached, placed in trays, and inoculated at the abaxial leaf surface with 10 µl droplets of a *P. infestans* zoospore suspension. Inoculated leaves were incubated at high humidity at 18 °C in the dark for the first 24 h, followed by a 16 h photoperiod. Lesion diameters were measured 6 days after inoculation (dai). Average lesion areas were determined as previously described (Vleeshouwers *et al.*, 1999), and normalized to those of TRV:*GUS*-treated control plants.

*Botrytis cinerea* isolate B05.10 was cultured on malt extract agar medium at 20 °C and sporulation was induced by UV light. Conidia were harvested from sporulating plates, resuspended in sterile water, and filtered through cheesecloth. Conidia were washed twice and resuspended in potato dextrose broth (PDB; 12 g l^–1^). *N. benthamiana* plants were inoculated with 2 µl droplets of 1 × 10^6^ conidia ml^–1^ and placed in closed transparent boxes at room temperature. Plants were kept in the dark for the first 24 h. Lesion diameters were measured 3 dai, and those expanding 3.5 mm were scored as secondary lesions.

*Pseudomonas syringae* was cultured on King’s B medium with rifampicin (100 µg ml^–1^) at 28 °C. Inoculum of *P. syringae* pv. *s yringae* isolate B728a (*Pss*) was prepared from an overnight culture that was resuspended in 10 mM MgCl_2_ to an OD of 0.01 [7 × 10^6^ colony-forming units (cfu) ml^–1^] and sprayed on detached *N. benthamiana* leaves placed in closed trays that were kept at 21 °C for 3 d. To quantify the total amount of bacteria, leaf samples were ground in 10 mM MgCl_2_, and colony-forming units were counted after dilution plating. For infiltration of *Pseudomonas syringae* pv. *tomato* (*Pst*) *ΔhrcC*, an overnight culture was collected and resuspended in 10 mM MgCl_2_. The inoculum concentration used for infiltration was set to an OD_600_ of 0.5 (~1.0 × 10^8^ cfu ml^–1^) (Kim *et al.*, 2011).

### Agroinfiltration and virus-induced gene silencing

*Agrobacterium tumefaciens* strains containing binary TRV vectors were grown in medium containing the appropriate antibiotics at 28 °C. *Agrobacterium* cultures were centrifuged and resuspended in infiltration buffer [per litre: 10 mM MES pH 5.6, 5 g of Murashige and Skoog (MS) salts (without vitamins), 20 g of sucrose, and 150 µM acetosyringone]. *Agrobacterium* strains harbouring pTRV2 derivatives or pTRV1 were mixed in a 1:1 ratio (at a final OD_600_ of 1.0), and VIGS was performed on 16-day-old *N. benthamiana* by agroinfiltration into the first two emerging leaves (Peart *et al.*, 2002). TRV:*GUS* and TRV:*SGT1* were included as controls (Tameling and Baulcombe, 2007). The fifth and sixth leaf were harvested 3–4 weeks after agroinfiltration, and subsequently used for further analysis.

### Quantitative RT–PCR

Total RNA was isolated from *N. benthamiana* leaves by a NucleoSpin RNA plant mini-kit (Clontech). cDNA synthesis was performed on 1 µg of total plant RNA using an oligo(dT) primer and M-MLV reverse transcriptase (Invitrogen). Quantitative reverse transcription–PCR (qRT-PCR) was performed using SYBR Green master mix (Promega), gene-specific primers (Supplementary Table S3), and 2 µl of 10-fold diluted cDNA using a Bio-Rad 7300 thermocycler. Gene expression levels were normalized to *Actin* expression (Gabriëls *et al.*, 2007).

### Callose deposition assays

*Nicotiana benthamiana* leaf discs collected 14 h after infiltration with *Pst* DC3000 *ΔhrcC* were cleared with ethanol and stained with aniline blue (1%, w/v). Callose deposition was visualized using epifluorescence microscopy as previously described (Bouwmeester *et al.*, 2011). Four or five leaf disks were examined for every sample, and three microscope pictures (3.5 mm^2^) were taken randomly per leaf disk. The total numbers of callose spots were counted per microscope picture.

## Results and Discussion

### Identification and phylogenetic analyses of exocyst subunits from Solanaceous plants

To identify exocyst subunit genes in Solanaceous plants, BLAST analyses were performed by using protein sequences of Arabidopsis exocyst subunits (listed by Chong *et al.*, 2010) as query against the predicted proteomes of *N. benthamiana*, potato (*S. tuberosum*), and tomato (*S. lycopersicum*). Genes for all eight known exocyst subunits were identified in *N. benthamiana*, tomato, and potato. Both tomato and potato contain one copy of *Sec6*, *Sec8*, and *Sec10*, two copies of *Sec3*, *Sec5*, and *Sec15*, three copies of *Exo84*, and multiple copies of *Exo70*; that is, 22 in tomato and 21 in potato versus 23 in Arabidopsis (Table 1). In contrast, *N. benthamiana* has twice the number of exocyst subunit genes (Table 1) as anticipated based on its allopolyploid genome (Goodin *et al.*, 2008).

**Table 1.** Predicted copy numbers of exocyst subunit genes in Solanaceous plants

	Arabidopsis	*N. benthamiana*	Tomato	Potato
*Sec3*	2	4	2	2
*Sec5*	2	4	2	2
*Sec6*	1	2	1	1
*Sec8*	1	2	1	1
*Sec10*	2	2	1	1
*Sec15*	2	4	2	2
*Exo70*	23	44	22	21
*Exo84*	3	6	3	3

**Fig. 1.**
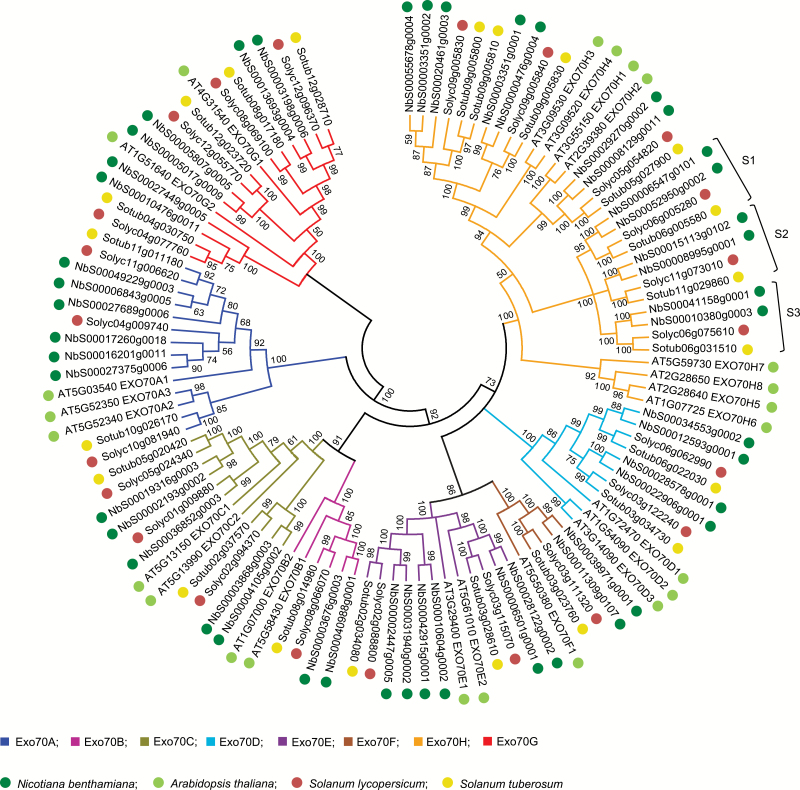
Phylogenetic tree based on predicted Exo70 proteins of Arabidopsis and the Solanaceous plants *N. benthamiana*, tomato (*S. lycopersicum*), and potato (*S. tuberosum*). Exo70 proteins cluster in eight subclades. Exo70H clusters S1, S2, and S3 lack Arabidopsis homologues and could be Solanaceous-specific. Protein sequences were aligned with ClustalW at default settings and phylogenetic analysis was performed using the Neighbor–Joining method with 5000 bootstraps in MEGA5. Branches corresponding to partitions reproduced in ≤50% bootstrap replicates were collapsed. (This figure is available in colour at *JXB* online.)

Phylogenetic analysis was performed to reveal the evolutionary relationships among the exocyst subunits from *N. benthamiana*, tomato, potato, and Arabidopsis. The phylogenetic tree in Fig. 1 shows that the Arabidopsis Exo70 proteins cluster with Solanaceous Exo70s into eight subclades (i.e. Exo70A–Exo70H), which is in line with previous phylogenetic analyses (Synek *et al.*, 2006; Chong *et al.*, 2010; Sekereš *et al.*, 2017). This clustering and the rather comparable number of *Exo70* genes in the analysed plant genomes indicate that the *Exo70* gene family was already largely present in the last common ancestor of Brassicaceous and Solanaceous plants. There is one branch in the Exo70 tree that only contains Exo70H genes unique for Arabidopsis (i.e. Exo70H5, -H6, -H7, and -H8). Three clusters do not comprise Arabidopsis orthologues, but solely contain Solanaceous-specific Exo70H subunits (i.e. Exo70H-S1, -S2, and -S3).

**Fig. 2.**
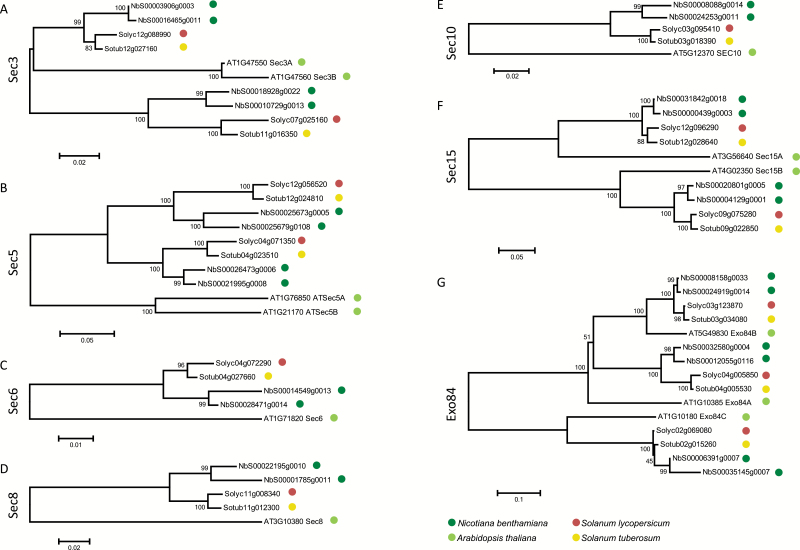
Phylogenetic trees based on the predicted exocyst subunits Sec3, Sec5, Sec6, Sec8, Sec10, Sec15, and Exo84 of Arabidopsis, *N. benthamiana*, tomato (*S. lycopersicum*), and potato (*S. tuberosum*). Protein alignments were constructed using ClustalW at default settings. Phylogenetic analysis was performed by the Neighbor–Joining method with 5000 bootstraps in MEGA5. Branches corresponding to partitions reproduced in ≤50% bootstrap replicates were collapsed. (This figure is available in colour at *JXB* online.)

The phylogenetic tree of Sec3 subunits shows that Arabidopsis Sec3A and Sec3B group together, whereas the Solanaceous Sec3 subunits are divided into two distinct subclades, with each containing one member of tomato, one of potato, and two of *N. benthamiana* (Fig. 2A). Also Arabidopsis Sec5A and Sec5B share high sequence similarity and cluster together in a manner comparable with that observed for Sec3 (Fig. 2B). As such, it is not possible to distinguish Solanaceous Sec3A from Sec3B, or Sec5A from Sec5B. It appears that duplication of Sec3 and Sec5 occurred twice independently, once in the lineage comprising Arabidopsis and once in the common ancestor of Solanaceous plants.

In contrast to Sec3 and Sec5, the two copies of Arabidopsis Sec15 (Sec15A and Sec15B) are not clustered but instead group with their homologues in Solanaceous plants (Fig. 2F). Similarly, the three Arabidopsis Exo84 paralogues were found to cluster with homologues from Solanaceous plants into three subclades. This indicates that duplication of Sec15 and triplication of Exo84 arose from an ancient duplication in a common ancestor of Brassicaceous and Solanaceous plants (Fig. 2G). The phylogenetic trees of the single-copy genes *Sec6* and *Sec8* show clustering of Solanaceous genes, as expected, with the Arabidopsis gene on a separate branch (Fig. 2C, D). *Sec10* was previously reported to be a single-copy gene in the Arabidopsis reference genome (Fig. 2E). However, Vukašinović *et al.* (2014), who re-sequenced the *Sec10* locus in Arabidopsis, revealed that it comprises two paralogous genes in tandem that are almost identical.

Our results are in agreement with the findings reported by Cvrčková *et al.* (2012), who analysed the evolutionary relationship of exocyst subunits from 10 different plant species, and distinguished three major groups. The first group includes the low copy number gene families Sec3, Sec5, Sec6, Sec8, and Sec10, which are duplicated in one or more plant species probably due to relatively recent gene duplication events that occurred independently in different plant lineages (Cvrčková *et al.*, 2012). The second group comprises the exocyst subunits Sec15 and Exo84. They are encoded by small gene families that emerged from a single ancestral gene. This expansion occurred much earlier during plant evolution than the gene duplications that shaped the first group. Similar to the second group, the third group comprising the multicopy Exo70 gene family with an enormous diversity among its paralogues probably evolved from ancient gene duplications in the common ancestor of land plants (Cvrčková *et al.*, 2012).

### Silencing exocyst subunit genes results in a range of developmental phenotypes

To study the function of the individual exocyst subunits in plant immunity, we made use of VIGS in *N. benthamiana* (Ratcliff *et al.*, 2001), preventing lethality issues upon null mutations (Zhang *et al.*, 2010). We generated VIGS constructs with a binary TRV vector as backbone that have the potential to silence paralogous exocyst subunit genes. For the subunits in the first major group—Sec3, Sec5, Sec6, Sec8, and Sec10—we were able to generate one construct per subunit that targets all paralogues in *N. benthamiana.* For the others—Sec15, Exo70, and Exo84—which have a lower sequence similarity, we needed multiple constructs per subunit to accomplish silencing of all subunit genes (Supplementary Table S2). Three weeks after treatment of *N. benthamiana* with the various exocyst TRV constructs, transcript levels of the targeted genes were found to be reduced in comparison with the levels in TRV:*GUS*-treated control plants (Supplementary Fig. S1). The silencing levels varied, and only three genes, namely *Exo70D1*, *Exo70E1*, and *Exo70H-S3*, showed a silencing efficiency of <40%.

**Fig. 3.**
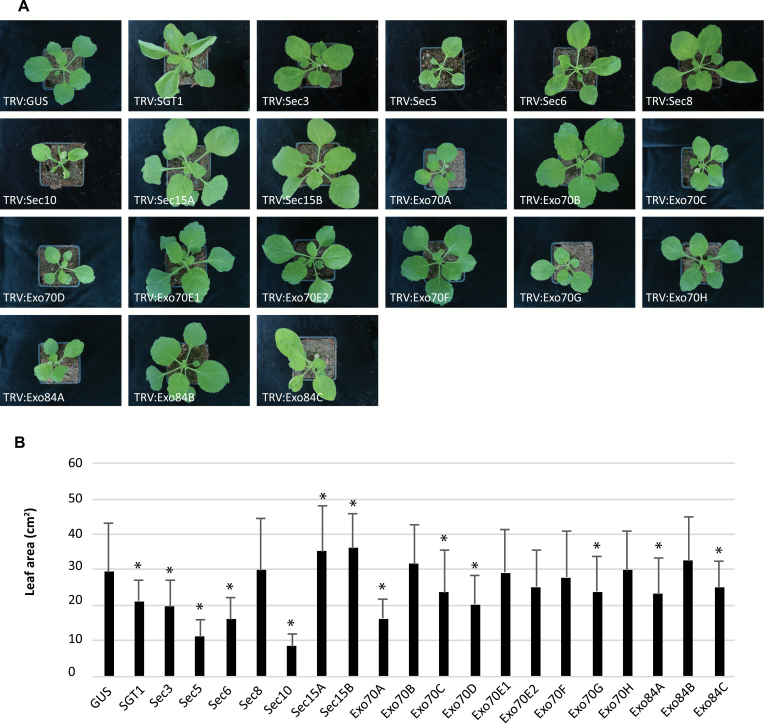
Developmental phenotypes. (A) Morphology of *N. benthamiana* plants upon silencing of exocyst subunit genes. Representative pictures were taken 3 weeks after infiltration with the TRV silencing constructs. Plants infiltrated with TRV:*GUS* and TRV:*SGT1* constructs were included as controls. (B) Surface area of leaves of *N. benthamiana* plants upon silencing of exocyst subunit genes. Surface areas of leaves of similar age from 3-week-old plants were measured and calculated using Image J software. Error bars indicate the SD in at least three independent biological experiments. Significant differences compared with TRV:GUS-treated control leaves are indicated by asterisks (*n*≥20 combined from at least three independent experiments, one-sided Student’s *t*-test; *P<0.05). (This figure is available in colour at *JXB* online.)

Three weeks after TRV treatment, we monitored changes in plant development. The results show that silencing of the exocyst subunit genes with low copy numbers (i.e. *Sec3*, *Sec5*, *Sec6*, *Sec8*, and *Sec10*) leads to aberrant plant development (Fig. 3). The whole plants and also individual leaves were smaller in comparison with the control (TRV:*GUS*), and in particular *Sec10*-silenced plants were found to be severely dwarfed. Also silencing of some members of the extended *Exo84* and *Exo70* gene family, namely *Exo84A*, *Exo84C*, *Exo70A*, *Exo70C*, *Exo70D*, and *Exo70G*, resulted in growth retardation, whereas silencing of the remaining members of these families did not cause any changes in growth morphology. In the case of *Sec15*, the leaves were larger in comparison with the control, which indicates that Sec15 is somehow involved in negative regulation of plant growth. Functional redundancy might explain the observed phenotypic differences among various *Exo70* family members. Dwarfism was also observed in mutant lines of the Arabidopsis exocyst subunit *Sec6*, *Sec8*, *Exo70A1*, *Exo70B1*, and *Exo84B* (Cole *et al.*, 2005; Synek *et al.*, 2006; Fendrych *et al.*, 2010; Kulich *et al.*, 2013; Li *et al.*, 2013, Wu *et al.*, 2013) and the moss *Physcomitrella patens* subunit gene *Exo70.3d*, which encodes an Exo70G subunit (Rawat *et al.*, 2017).

### Exocyst subunits play a role in plant defence against *Phytophthora infestans*

**Fig. 4.**
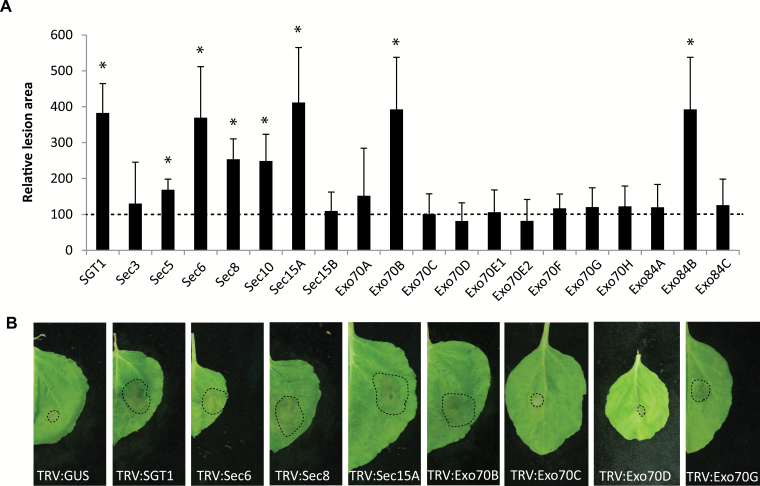
The majority of exocyst subunit gene-silenced *N. benthamiana* plants show enhanced susceptibility to *P. infestans*. (A) Lesion diameters were measured at 6 dai with *P. infestans*, and average lesion areas were calculated and normalized to the control TRV:*GUS*, which is set at 100. Error bars indicate the SDs. Significant differences compared with the control are indicated by asterisks (*n*≥12, one-sided Student’s *t*-test; *P<0.05). Experiments were performed in multiple batches, and repeated at least three times with comparable outcomes. (B) Examples of lesions caused by *P. infestans* isolate 14-3-GFP on *N. benthamiana* leaves silenced for various exocyst subunit genes. Pictures were taken 6 dai. (This figure is available in colour at *JXB* online.)

Previously we showed that Sec5 plays a role in plant defence against the oomycete pathogen *P. infestans* (Du *et al.*, 2015). To investigate whether other exocyst subunits are required for defence against this hemi-biotrophic pathogen, we first silenced the exocyst subunit genes in *N. benthamiana* and subsequently inoculated the plants with *P. infestans* isolate 14-3-GFP. Six days after inoculation, plants silenced for *Sec5*, *Sec6*, *Sec8*, *Sec10*, *Sec15A*, *Exo70B*, and *Exo84B* showed significantly larger lesions compared with the TRV:*GUS*-treated control plants (Fig. 4), thus pointing to increased susceptibility to *P. infestans*. In contrast, silencing of *Sec3*, *Sec15B*, *Exo70A*, *Exo70C*, *Exo70D*, *Exo70E1*, *Exo70E2*, *Exo70F*, *Exo70G*, *Exo70H*, *Exo84A*, and *Exo84C* did not cause significant changes in lesion sizes in comparison with the control. Apart from Sec3, all low copy number exocyst subunit genes seem to be required for defence against *P. infestans*. This is in contrast to the expanded *Exo70* gene family of which only subclade Exo70B seems to be involved in defence against *P. infestans*. In other studies, subclades Exo70F and Exo70H1 were found to be required for penetration resistance against *B. graminis* in barley and Arabidopsis, respectively (Pečenková *et al.*, 2011; Ostertag *et al.*, 2013). Moreover, Exo70H1 also plays a role in immunity against the bacterial pathogen *P. syringae* (Pečenková *et al.*, 2011). The fact that we found no indications for a role for these subclades in *Phytophthora* resistance suggests that plants have multiple exocytotic pathways, with each pathway operating in defence against a subset of pathogens or classes of pathogens. As hypothesized by Žárský *et al.* (2013), these exocytotic pathways might be mediated by different Exo70 subcomplexes.

### Exocyst subunits are required for plant defence against the bacterial pathogen *Pseudomonas syringae*

**Fig. 5.**
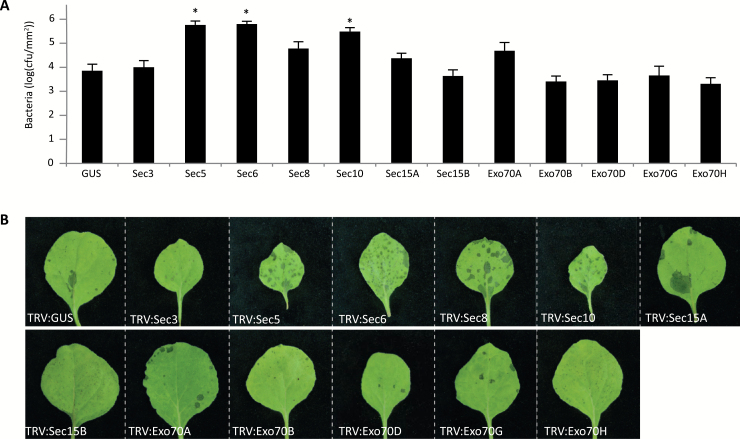
Response of exocyst subunit-silenced *N. benthamiana* plants to *Pseudomonas syringae.* (A) Bacterial growth was measured at 3 dai. Error bars indicate the SD. Significant differences compared with the control TRV:*GUS* (*n*=4, one-sided Student’s *t*-test) are indicated by asterisks (*P<0.05). Experiments were repeated three times with comparable outcomes. (B) Leaf spots caused by *Pss* isolate B728a on *N. benthamiana* leaves silenced for various exocyst subunit genes. Pictures were taken 3 dai. (This figure is available in colour at *JXB* online.)

We also investigated whether exocyst subunits are required for defence against the biotrophic bacterium *Pss*. We selected 12 exocyst subunits based on published data (Pečenková *et al.*, 2011; Stegmann *et al.*, 2012, 2013; Kulich *et al.*, 2013; Zhao *et al.*, 2015) and on the phenotypes that we observed upon infection with *P. infestans*. TRV-treated *N. benthamiana* plants were spray-inoculated with *Pss* isolate B728a and bacterial growth was checked 3 dai. As shown in Fig. 5, plants silenced for *Sec5*, *Sec6*, or *Sec10* showed a significant increase in bacterial growth compared with the TRV:GUS-treated control plants, whereas silencing of *Sec3*, *Sec8*, *Sec15A*, *Sec15B*, *Exo70A*, *Exo70B*, *Exo70D*, *Exo70G*, or *Exo70H* did not cause significant differences in bacterial infection. Others reported that Arabidopsis Exo70B and Exo70H mutants showed enhanced susceptibility to *Pst* (Pečenková *et al.*, 2011; Stegmann *et al.*, 2012, 2013; Zhao *et al.*, 2015), but in our assays we found no indications for a role for *N. benthamiana* Exo70B and Exo70H in resistance to *Pss*.

### Several exocyst subunits facilitate host susceptibility to the necrotrophic fungus *Botrytis cinerea*

**Fig. 6.**
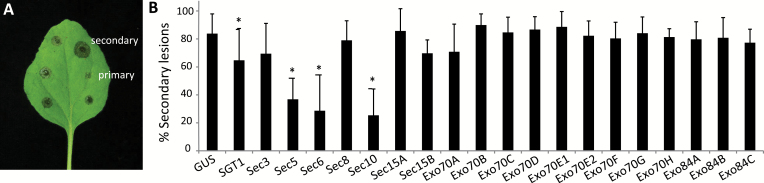
Silencing of exocyst subunit genes in *N. benthamiana* reduces susceptibility to the fungal pathogen *B. cinerea*. (A) Primary and secondary lesions 2 dai with *B. cinerea* isolate B05.10. (B) Percentage of secondary lesions 3 dai with *B. cinerea*. Error bars indicate the SD of at least three biological replicates. Significant differences with the control TRV:*GUS* (*n*≥3 experiments, one-sided Student’s *t*-test) are indicated by asterisks (*P<0.05). Each experiment contained at least 50 lesions per silenced exocyst subunit. (This figure is available in colour at *JXB* online.)

To explore the role of the exocyst subunits in defence against necrotrophic pathogens, we performed infection assays with the broad host range fungus *B. cinerea*. TRV-treated *N. benthamiana* plants were inoculated with spores of *B. cinerea* isolate B05.10. As early as 2 dai, two types of necrotic lesions could be discriminated, namely halted primary lesions and expanding secondary lesions (Fig. 6A; Van Kan., 2006). At 3 dai, ~80% of the inoculated spots on leaves of TRV:GUS-treated control plants developed into secondary lesions, and similar percentages were found on the majority of exocyst-silenced *N. benthamiana* plants. None of the exocyst-silenced plants was found to be more susceptible to *B. cinerea*. *SGT1*-silenced plants showed fewer secondary lesions, and this is in line with the results reported by El Oirdi *et al.* (2007). In our assays, we observed significantly fewer secondary lesions on plants silenced for *Sec5*, *Sec6*, or *Sec10* (Fig. 6B). These results are in clear contrast to the enhanced susceptibility that we observed towards biotrophic pathogens. This suggests that these exocyst subunits are, on the one hand, required for defence against biotrophic pathogens, and, on the other hand, contribute to colonization by the necrotrophic fungus *B. cinerea.* This apparent dual role of exocyst subunits in defence might be related to the function of the exocyst complex in endomembrane cycling of cell surface receptors. Receptors that recognize PAMPs of *B. cinerea* can induce necrotic responses, as shown in the case of the Arabidopsis RBPG1 receptor (Zhang *et al.*, 2014), and this is probably favourable for a pathogen that thrives on dead plant cells. On the other hand, receptors that recognize PAMPs of biotrophic pathogens often induce pattern-triggered immunity (PTI), thereby creating an unfavourable condition for these type of pathogens.

### Exocyst subunits are involved in callose deposition

**Fig. 7.**
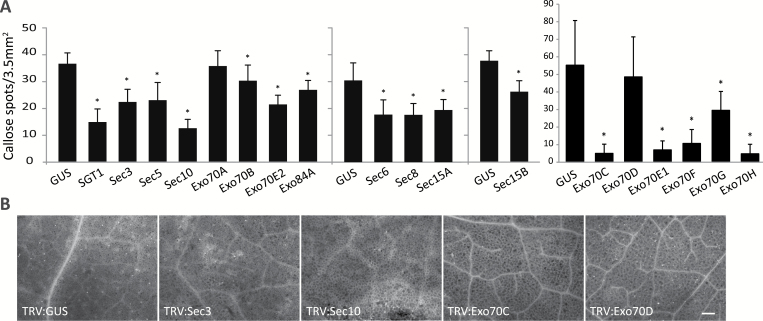
Exocyst subunit genes are required for callose deposition. (A) Callose deposition in *N. benthamiana* leaves 16 h after infiltration with *Pst* DC3000 Δ*hrcC.* The *y*-axis shows the amount of callose spots per microscopy image (i.e. 3.5 mm^2^). Error bars represent the SE, and asterisks mark significant differences relative to TRV:*GUS* (*n*≥9, one-sided Student’s *t*-test; *P<0.05). Experiments were performed in multiple batches each containing TRV:*GUS*-treated plants as control. (B) Representative microscopy images showing callose deposition as bright spots upon aniline blue staining. Scale bar=200 μm.

Callose deposition is a process that probably depends on the exocyst complex. This is exemplified by the fact that Sec5 is required for proper callose deposition during plant defence (Du *et al.*, 2015). Hence, we wondered whether other exocyst subunits play a role in callose deposition. To test this, leaves of silenced *N. benthamiana* plants were infiltrated with *Pst* Δ*hrcC* to elicit callose deposition. Microscopic analysis of leaves stained with aniline blue revealed that callose deposition in *SGT1*-silenced plants was significantly reduced compared with TRV:GUS-treated control plants (Du *et al.*, 2015). Callose deposition was also significantly reduced in most exocyst subunit gene-silenced plants, with the exception of Exo70A and Exo70D (Fig. 7). This suggests that the latter two are not required for callose deposition and this could be explained by the diversification of the Exo70 family where specific subunits mediate different exocytotic processes. The reduced callose deposition in most exocyst-silenced plants is in line with the findings by Pečenková *et al.* (2011) that Exo70B2 of Arabidopsis is required for proper formation of papillae, of which callose is a major component, and by Kulich *et al.* (2015) that Arabidopsis Exo70H4 plays a role in callose deposition in trichomes. Exocyst subunits may have a role in transporting callose synthases to the plasma membrane, and this may explain why callose deposition is disturbed in leaves lacking one of the subunits of the exocyst. Notably not all exocyst subunit gene-silenced plants that show reduced callose deposition have aberrant phenotypes in the disease assays (Supplementary Table S4). This could be due to the fact that the pathogen strains that we used are not virulent enough to breach defence barriers other than callose deposition or, alternatively, that the strains are too virulent to see differences in disease phenotypes.

## Conclusion

In this study, we showed that several exocyst subunits function in exocytosis-related processes, including plant development, plant defence, and callose deposition. We found a role for three exocyst subunits (i.e. Sec5, Sec6, and Sec10), in defence against all tested pathogens. However, their role changes depending on the type of pathogen that elicits the defence response. They either contribute to defence by raising a barrier against the (hemi-)biotrophic pathogens *P. infestans* and *Pss*, or they help the necrotrophic pathogen *B. cinerea* to colonize. Sec8, Sec15A, Exo70B, and Exo84B were only found to function in defence against *P. infestans*. Our study further showed diverse roles for Exo70 and Exo84 paralogues in plant development and defence, which supports the idea that these clades have evolved in such a way that each paralogue has a specialized function, either in exocytosis or in other cellular processes. The present study provides insights into the roles of Solanaceous exocyst subunits in defence against three unrelated pathogens. It forms a platform for further functional studies, in particular on the role of Solanaceous exocyst subunits in immunity and autophagy, as well as the role of salicylic acid and other defence-related hormones in exocyst-mediated defence responses.

## Supplementary data

Supplementary data are available at *JXB* online.

Fig. S1. Relative expression of exocyst subunit genes in silenced *N. benthamiana* plants 3 weeks after TRV treatment.

Table S1. Gene IDs and protein sequences of exocyst subunits of *N. benthamiana*, tomato (*S. lycopersicum*), and potato (*S. tuberosum*).

Table S2. The TRV silencing constructs used in this study and the sequences of the fragments inserted in the binary vector pTRV2.

Table S3. Primers used for qRT–PCR.

Table S4. Summary of phenotypic characteristics of *Nicotiana benthamiana* plants in which the various exocyst subunit genes are silenced.

Supplementary Figure S1

Supplementary Table S1

Supplementary Tables S2-S4

## Abbreviations:

BFA: brefeldin-A
dai: days after inoculation
NLR: nucleotide-binding leucine-rich repeat
PAMP: pathogen-associated molecular pattern
*Pss*: *Pseudomonas syringae* pv. *syringae*
*Pst*: *Pseudomonas syringae* pv. *tomato*
ROS: reactive oxygen species
SGN: Sol Genomics Network
TRV: *Tobacco rattle virus*
VIGS: virus-induced gene silencing
