# Supplementary material for: Solanaceous exocyst subunits are involved in immunity to diverse plant pathogens
Source: J Exp Bot. 2018 Jan 10;69(3):655–66. doi: 10.1093/jxb/erx442 (PMC5853398; doi:10.1093/jxb/erx442)
Supplement: Supplementary Figure S1 [file erx442_suppl_supplementary-figure-s1.pdf]

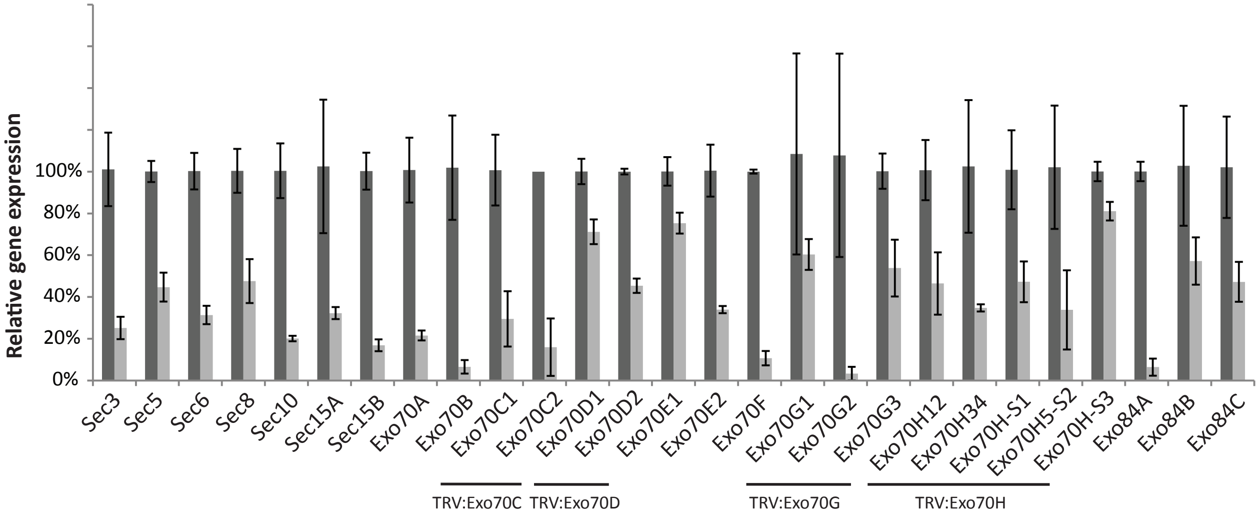

Figure S1. Relative expression of exocyst subunit genes in silenced *N. benthamiana* plants three weeks after TRV treatment. Transcript levels were determined by Q-RT-PCR and normalized to *Actin*. The bars show the mean fold changes in transcript levels (± standard deviation) relative to the transcript levels in TRV:*GUS*-treated control plants, which were set at 100%. (▮, TRV:*GUS*; ▮, TRV:*exocyst subunit*). Four of the silencing constructs target multiple genes in one (sub)clade (indicated by the horizontal lines) but expression of each gene in these (sub)clades is monitored separately by using gene-specific primers in the Q-RT-PCR.
