# Supplementary material for: Solanaceous exocyst subunits are involved in immunity to diverse plant pathogens
Source: J Exp Bot. 2018 Jan 10;69(3):655–66. doi: 10.1093/jxb/erx442 (PMC5853398; doi:10.1093/jxb/erx442)
Supplement: Supplementary Tables S2-S4 [file erx442_suppl_supplementary-tables-s2-s4.pdf]

Table S2. The TRV-silencing constructs used in this study and the sequences of the fragments inserted in the binary vector pTRV2.

Construct	Insert
**TRV:Sec3**	GAATTCCTTCAAAACATTGAGGCATGTGAGTGGTTAACTAATGCTTTACGTGGTCTTGAAGCGCCTAATTTGGATCCTAGCTATGCAAACATGAGAGCTGTAAAGGAGAAACGATCTGAGCTTGATAAGTTAAAAACAACATTTGTTCGAAGAGCATCTGAGTTCTTGAGAAACTATTTTACCAGTTTGGTGGATTTTATGATAAGTGACAAGAGTTATTTTTCTCAGCGTGGACAATTGAAGAGGCCTGACCATGCTGATCTGAGATACAAGTGCAGGACATATGCTCGTCTTCTGCAGCATTTGAAGAGTC
**TRV:Sec5**	TTGCTAATCGTGCTTTTGGGCCACTATTTGAAAGACAGGCTCAAGCTGAGAAGATAAGATCTGTTCAAGGAATGCTGCAGAGGTTCCGAACACTATTTAACTTGCCCAGCACGATTCGTGAGAGCATTAGTACTGGTGAATATGACCTAGCAGTCAGGGAGTACAGGAAAGCGAAGTCAATCGTTCTGCCTTCTCATGTTGGAATTTTGAAGCGCGTGCTTGAGGAGGTTGAAAGAGTTATGCAAGAATTTAAAAGCAAGCTTTATAAGTCTCTGGAAGATCCTCAAATTGACCTAACAAATCTTGAAAATAATGTGAGACTCTTGTTGGAGTTGGAACCCGAATCAGATCCTGTGTGGCACTATTTGAATATACAGAACCAGAGAATCCGAGGTTTGCTTGAGAAGTGCACTTTGGATCATGAAGCAAGGACGGAAAATTTCAGGAATGAGATGCGTGAAAGAGCTTTATCTGATGCAAAATGGAGGCAAATTCAGCAAGATTTGAACCATTCTTCAGATGTTGATTACTCTGATTCTCATGAAAATACATACTTGCCGGGTGATTCACAACAAGT
**TRV:Sec6**	TTGACAAGAATTGCTTGGGCCCTTTAAGAAAAGCTTATTGCACATCCCTTAATTTGCTTTTACGCCGGGAGCTCTGGAGCTAAGTTCTAGTACTCTTGAAGCTCTCCCACAGAACTATGCCGAGCAGGTTAATTTCGAGGACACATGCAAAGGTTTTTTAGAGGTTGCAAAGGAAGCTGTTCATCAAACTGTCAGTGTCATCTTTGAAGATCCAGGAGTAGAGGAATTGCTCGTTAAGTTGTATCAGAAAGATTGGTTGGAAGGACAGGTTACCGAGTTTTTAGTTGCAACATTTTCAGACTACTTCACCGATGTTAAAATGTACATTGAAGAAAGATCATTTAGGCGTTTTGTCGAGGCTTGCCTGGAGGAGACTGTTGT
**TRV:Sec8**	AGAACCATGTCATTGTTGGAGAACTTCTTGAGTCAAAGTGCTCGCAACAAGTTGACTTGAACACACCTAAGTCCATGCCTACAGACATCAGTTGGAACCCTGATTCAGATGCATCTCGTGACACTGGAGGCTATAGTATTGGCTTTTCATTGACCGTCTTACAGAGTGAATGCCAACAACTCATATGTGAGATCCTGCGAGCTACTCCAGAAGCTGCATCCGCAGATGCTGCTG
**TRV:Sec10**	GAATTCGCACCGCAAGACACTTTCTGAGCTGGAAAAAGGTGTAGACGGCTTGTTTGATAGCTTTGCACGGTTGGATTTGCGTATATCTAGTGTGGGTCAAACAGCTGCCAAAATAGGAGATCATCTGCAGAGTGCAGATGCTCAGAGAGAAGTTGCTAGTCAGACAATAGAGCTCATAAAGTACTTGATGGAGTTCAATAGCAGTCCAGGAGACCTGATGGAACTTTCTCCATTGTTTTCTGATGATAGTCGTGTTGCTGAAGCTGCTTCGATTGCTCAAAAATTAAGATCATTTGCTGAGGACGATATTGGAAGGGATCC
**TRV:Sec15A**	AGATCTAGGTCCTATGGTCAGGCTTTCTTTTGAAAGCGGGAAGCCTGATGCCCTTCTACAACAGCTTAAAAATGTCGTCAAAAAGAAAGAAGTTGAAATAGAGGAGCTTTGTAAGCTTCACTATGAGGAATTTATTGTTGCAGTGGATGAGCTGCGCGGTGTCTTGGTTGATGCAGAAGAGCTAAAAGCTGAGTTGCAAACTGATAATCTGAAGCTGCAAGATGTTGGGAGTACCCTTCTTTTAAAACTTGAAGAGTTTCTTGAATCTTATTCAATTAAGAAAAATGTTACAGAAGCGATTAAGATGTCAGGCAACTGTGTCCAAGT
**TRV:Sec15B**	TGCCTGCATTTCCTTATGTTGCACCGTTTTCGTGTACGGTGCCTGATTGCTGTAGAATAGTGCGGTCCTTTATCGAGGATTCAGTAAGTTTTATGTCACATGGTGGTCAGCTTGATTTCTATGATGTGGTGAAGAAGTACTTGGATCGGCTTTTAACTGAAGTCCTGGATGGAGCCCTGTTGAAGCTCATTCATACGTCTATCGGTGGTGTTACCCAAGCAATGCAGATGGCAGCTAACATGGCTGTGTTTGAACGCGCTTGTGATTTTTTCTTTCGTCATGCTGCACAGCTTTCAGGGATTCCATTGA
**TRV:Exo84A**	ACAGGCAATCCCGTCCTATAGAGCAGAGAGAACTGAAACGGAGACTCCAACGTTTAGTTGATCAGCTCCGTGACAGCTTTTGTAGGCAACATGCACTTGAACTCATTTTCTTGGAAGATGGTGGCGTTCGTTTAAGTCCAGACATGTACCTAAATATGGAAGGAAGTCCAGAAGAGATAGAGTGGTTTCCATCTCCAGTATATCAGGAAATGTTTGAGAAGTTGACTCGAATTGCTAGCATAGCATCAGACATGTTTGTGGGAAGAGAAAGATTTGCTACTATTCTTTTGATGAGACTCACTGAAACTATCA
**TRV:Exo84B**	AGGCTGGCGATTAGCGAACCAACAACACAATCAAACAAATTTTTATTCTCTTTCTACGAACAACTAATAATCTCCTCTTCTTTCGTCAATCATGGCGATGGCGATAACAACTCTCACCTTAGGATTAGATCTACCCTTTTGATTTTCTTACGTCTTCCCTATTTTTGCTCTATATTTCAACCCTAAACATCCGCCATTAAGATTCACCTCATGGCTTCCGTCAAATCCTCTCGTTCAAGAGCTCATGCGGTCACTCAGTCTAAAGGAATCAACAAGGATACCGGCCCCAAGCTCGAAGAAAATCTTAACGTCTTCAAATCCGACAATTTCGATGCTGATGCTTTTGTCCAGTCCAAATGTCATTCTCTCAACGAGAAGGAAATAAGGCAGCTGTGCTCTTATCTATTGGAACTGAAAAGGGCTTCTGCTGA
**TRV:Exo84C**	GAATTCTAGTCAAAGAAATAGTGGAGAAGCTCACCCAGTTAGTCATTCTACATTTTGGAGCTAACATATTGACCAGAATTTCACATCTTTTTGACAAGTATGTGGATTCTTTGATTAAAGCCCTGCCTGGTCTTTCTGAGGATGATAATCTCACTGAGCTGAAAGAACCTGTTCTTTTTAGAGCTGAAACAGACTCGGAGCAGCTTGCTCTATTGGGAACTGCATTTACCATTGCTGAGGAACTATTGCCTATGGTTGTATCTAGAATTTGGAATGTTCTTAATGAAAGCAAGGAAGCGAGCTC
**TRV:Exo70A**	ATTCATTTTATGCGGATTGCTGTCAAATTGTTATTTGCTGCGGAGCGAAAAGTTTGTGATCAAATGTTTGAAGGATTTGAGCACCTCAAAGATCAATGTTTTGCTGAAGTTACTACTGGGAGTGTGGCTGTGCTGCTTAGTTTTGGTGATGCAATTGCAAAAAGCAAAAGATCACCTGAGAAGTTGTTTGTGCTTCTAGATATGTATGAAATAATGCGGGAACTTCATTCAGAGATTGAATCGCTTTTCATAGGTAAAGCTTGCAATGAAATTAGGGAATCTGCCTTTGGTTTGACA
**TRV:Exo70B**	TGTTTATGAGGCGCTTAGGGATTTGATGCCTGAATTTGAATTGTTGTTTATGGATCAATACTGTGTGTTATTGAGGAATGAAGCTTTGACTATATGGAGAAGATTAGGTGAGGCTATTAGAGGGATTTTTATGGATTTGGAGAATTTGATTCGCCGTGATCCTGCCAAAACGCCTGTTCCAGGTGGTGGACTCCACCCGATTACTCGATACGTGATGAATTATCTCCGCGCAGCTTGTCGATCCCGGATTTCTCTTGAACAGGTTTTTGAGGAGTCTGCTAGTGTTGTTGATTATAGAGGAGAAGTGGATGATAGAGCGCTTTCATCGTCTCCCTTGGCTGTTCAAATGGCGTGGACCATGGAGTTACTTGAGAGTAATCTGGA
**TRV:Exo70C (targets C1 and C2)**	AATTCGAGTACAAATATGCTTATTCAATCAGCCGCGTTGATGGTGTTCTTCAACGCGCAATGTCCTGAGAAAATCTTCAGCAATTTGTCTCGAGGTATGATGATCCAACTCCTTAATTTCGCTGAAGCCGTTTCAAGTGATGGGCGATACGTGGTGTAGGAGACGATCAACGATCGTTAGACATTACCACAAGAATTACCAAAGGGAAACATGGGGTAAGGTGCTCCAAATCAATGGGAAAGTGACAAAGCCAGTAGTGAAAGAGAGGTTCAAGAATTTCAGCACAATGCTCGACGAAATCCATCGTACGCAGAGCACTTGGGTGAGCTC
**TRV:Exo70D (targets D1 and D2)**	GAATTCCAGTTATCGATCTACTAACAGCATTCGTGAAGTCGATCTCATGCCGTCAGAAGCAATATACGATCTCCGGTGCATCGCGGAGCGAATGATTTCAGCTGGATATCTCCGGGAGTGTATTCAGGTGTATGGCAGTGTACGCAAGTCTGCTGTGGATTCGAGCTTCCGGAAGCTTGGAATAGAGAAGCTGAGCATAGGAGATATACAGAGACTTGACTGGGAGACTCTCGAAGCAAAGATTCGTAGATGGATACGAGCAGCAAAAGTATGCCTTCGCATACTTTTCGCTAGCGAGAAGAAGCTCTGTGAGCAAATGAGCTC
**TRV:Exo70E1**	GAATTCTGATGAACAACATCCATTACATGGTTCAGAAAATCAAGAACTCCAAAATTAGAACTTATTTTGGTGATGAGTGGATAAAAACACATATTGTAAAATACGTGCAGCACGAAAAAAGCTACGAGAGAATAACATGGAGTTCAATCATATCTTTGATAACCGGTTATGAGAAGTTAGGGAAGGCAGTTCTGAAGGAGAGGTGCAGAAATTTCAGTATTGCTTTTGAGGAAGTGTATAAAAACCAGACAGGATGGACTATTCCAGATATTGAGAGCTC
**TRV:Exo70E2**	GCATTTATCAATGTCAGAAAAGATGCCTTGGACGATTGCCTCTTCATTCTTGAAGTTGAGAGGTTGAGCATTGAGGATGTACTGAAGATGGAATGGAACTCGTTGAACTCCAAAATCAGGAGGTGGATACGGGCTATGAAGATCTTTGTGCGCGTTTATCTTGCCAGTGAAAAGTGGCTAAGTGATCAGATTTTTAGCGAGCTGGAAGCAGTTAGTTCAGTTTGCTTTGCTGAGGCCAAAAGCTTCAATCGTACAGCTTCTGAACTTTGGTGAAGCCATAGCTATTGGCCCTCATCAACCGGAGAAGTTGATTCGGATTCTTGACATGTATGAGCTGCTCGCAGATCTTATCCCAGATATTGATGCTATGTACTCTGA
**TRV:Exo70F**	GAATTCATTGATCTGAGAGAGATTGCTAACCGTATGATTCGATCTGGATATGAAAAGGAATGTTGTCAAGTGTACTCTAGTGTTCGTAGAGAGGTGCTTGATGAGTGTTTAGCAATTCTTGGCATTGAGAAACTGAGCATTGAGGAGGTCCACAGGATTGAATGGCAGTCGCTGGACGAGAAAATGAAGAAATGGATTTATGCTGTAAAAGTTCTGGTGAGAATCCTTTTATCTGCTGAAAAGAGCTTGTGTGAACAGATTTTTGTAGGTTCTGAGTTGATTAAAGAAGTGTGCTTTATGGAAACTGCAAAGGGCTGTGTGAGCTC
**TRV:Exo70G (targets G1-3)**	GAATTCGACAAGATAATGGCTGTCTTCTTTAGATTCGGGGAAGGGGTTGCACGAAGTAAGAAAGAACCCCAAAAGCTGTTCAAGCTGTTAGATATGTTTGAATCATTGGAAAGGCTAAAATGGTAGTGTTCCGAGGCTGGTTAACTTTGTCAGTGAGTATTGTAATCAGATTCTTGAGGATGAATATTGGTCCACACTTATCCAAGTTGTGGAGATACATCAAAGTTTGCAGGCACTTAATTTGGATTACCTTGAGATTTCAGTCTCCGAGTTCAATGACGTGCAGAGCATAGAGGGTCACATTGCTCAGTGGGGTAAGCATTTAGAATTTGCTGTAAAGAGCTC
**TRV:Exo70H (targets H1-4 and S1-3)**	GAATTCAAACTTGATGCAAAAGGCTATGAAAAGACTTGAAAATGAATTTTACCAAATTTTGTCAACAAACAAGGAGCATTTAGATCCAGAATCAGTATCTAAGTCTAGTCGTTAGGTGAAAATTGGATATCAAAACAGGAGGGAAAAATCAAGCAGTTTGCATCAAACTATGAACGGCTAGGATGGAGCCACGTCATTGAATCTCCCTTTTATGTAAACTCGATGGTAAAGCACAACTTTACAAGGACGTTTCATTATCCTATCTGTTCTTAGCGAACAACTTAAACTATGTCGTTTCAGAGCTC

Table S3. Primers used for qRT-PCR.

Primer name	Sequence (5'-3')	Primer name	Sequence (5'-3')
Sec3-rtF	AGTTTGCAAACGAGCTCCGTG	Exo70D1-rtF	TCCCAGCTTATAGATCATTTCTC
Sec3-rtR	ACTCTCATCCACAAGTAGAGG	Exo70D1-rtR	CTGAGATCTTCTTCTTATGTGTTGTG
Sec5-rtF	AGTTAAGACGGATTGAGGAAGACC	Exo70D2-rtF	CATGAACTATATAAGTTTAATCTCGG
Sec5-rtR	TAGTGGCCCAAAAGCACGATTAGC	Exo70D2-rtR	GACAATAATCCAAATCAAATGAAGC
Sec6-rtF	TCATGAATGCGTATGTTGAGAGAATG	Exo70E1-rtF	GGCCAAGCTGTAGCTATTGG
Sec6-rtR	CTCGTACAATTTGCACCTGCT	Exo70E1-rtR	TGCAGAATCTCCAAGATTTTTCA
Sec8-rtF	ATATACCGGCCTGTTCTTCAG	Exo70E2-rtF	AACAGGCAGCCTTTTGAGC
Sec8-rtR	TGTACAGCTTTCCGATAATCAAC	Exo70E2-rtR	AACATCAAATTGGCAATGCATC
Sec10-rtF	ACACCACCAGATGCAGGAA	Exo70F-rtF	ATGCAGCTCTTGAATTTTGGAG
Sec10-rtR	TCTTGGACAGCAACTTCCTTC	Exo70F-rtR	TTCTCCTAGGCCATCTAACAC
Sec15A-rtF	TGCTATCAAGGCTATTGATCTG	Exo70G1-rtF	ACCAAAGTCTTCTTTGAGCT
Sec15A-rtR	AGTTTGCCCAATATCCTTTGC	Exo70G1-rtR	TCCAGCTTTCCATATTTGTTC
Sec15B-rtF	TGAAGAGATGCTTTCAGGTCTG	Exo70G2-rtF	AACCATTGGTACTTGTGCAAG
Sec15B-rtR	TCTCTTCAGAACTTGAACAGG	Exo70G2-rtR	TTAACTACTTGTCGATCAATGGC
Exo84A-rtF	TCTGAAGATCAAAATTTCTGGGAAG	Exo70G3-rtF	GAAACGATTAAAAGCTTTTAATG
Exo84A-rtR	AGCAACTGCTTCAATAGCTCTGC	Exo70G3-rtR	TCCTTCTTGTTCAACCAAC
Exo84B-rtF	TGCTCCTTAATGCACATTACCAG	Exo70H12-rtF	AACTATATGCAACAAACTATG
Exo84B-rtR	TGTATAAGCTGGCTCTTTACC	Exo70H12-rtR	ACTTTGATCTCGTCTCGTAG
Exo84C-rtF	AGAATCAGCTGGTTGAGATC	Exo70H34-rtF	ACATCATTCTACTTGCTGATTAC
Exo84C-rtR	AGTCTCGGGATAACAAGGAC	Exo70H34-rtR	AGTTTGCAGAGAAGAATGAGAATC
Exo70A-rtF	AGCAGTTGAGAAGGATGCAAC	Exo70H-S1-rtF	TAACTCGATACGTCATGAACTACC
Exo70A-rtR	AGCTGTGAATTTGAATCTCCTCC	Exo70H-S1-rtR	ACAACAGAAGGCGGAGAGTCC
Exo70B-rtF	TGATGAATAATGAAAGATACATTGTTC	Exo70H-S2-rtF	TGACTCGGTACGTTATGAATTATCTCG
Exo70B-rtR	ACTTAAGCTTTTCTTTCAAAGACC	Exo70H-S2-rtR	CTAGCCACGCAAGGCGGAC
Exo70C1-rtF	TGATGAACAACGGGCGATAC	Exo70H-S3-rtF	TAACTCGCTACGTCATGAACTACCTT
Exo70C1-rtR	ATTGCAACAGTTTACCCCAAG	Exo70H-S3-rtR	GATAAGCCAAGCGAGCCGTAC
Exo70C2-rtF	TTTGAAACAGAATGTTGTCAAGT	Actin-rtF	TATGGAAACATTGTGCTCAGTGG
Exo70C2-rtR	GTCTCTTTTCGCCAGGGAACAGAGT	Actin-rtR	CCAGATTCGTCATACTCTGCC

Table S4. Summary of phenotypic characteristics of *Nicotiana benthamiana* plants in which the various exocyst subunit genes are silenced.

Exocyst subunit	Level of silencing[a]	Leaf size	*P. infestans*	*Pss*	*B. cinerea*	Callose deposition
**Sec3**	<30	smaller	N	N	N	L
**Sec5**	30-50	smaller	S	S	R	L
**Sec6**	30-50	smaller	S	S	R	L
**Sec8**	30-50	N	S	N	N	L
**Sec10**	<30	smaller	S	S	R	L
**Sec15A**	30-50	larger	S	N	N	L
**Sec15B**	<30	larger	N	N	N	L
**Exo70A**	<30	smaller	N	N	N	N
**Exo70B**	<30	N	S	N	N	L
**Exo70C**	C1: <30<br>C2: <30	smaller	N	nd	N	L
**Exo70D**	D1: <90<br>D2: 30-50	smaller	N	N	N	N
**Exo70E1**	<90	N	N	nd	N	L
**Exo70E2**	30-50	N	N	nd	N	L
**Exo70F**	<30	N	N	nd	N	L
**Exo70G**	G1: 50-60<br>G2: <30<br>G3: 50-60	smaller	N	N	N	L
**Exo70H**	H12: 30-50<br>H34: 30-50<br>HS1: 30-50<br>HS2: 30-50<br>HS3: <90	N	N	N	N	L
**Exo84A**	<30	smaller	N	nd	N	L
**Exo84B**	50-60	N	S	nd	N	nd
**Exo84C**	30-50	smaller	N	nd	N	nd

[a] percentage of the expression level in control plants
S = more susceptible than control plants
R = more resistant than control plants
N = no significant difference with control plants
L = less callose deposition than in control plants
nd = not determined
